# Empirical assessment of functional somatic disorder (FSD): frequency, applicability, and diagnostic refinement in a population-based sample

**DOI:** 10.1186/s12916-025-04042-w

**Published:** 2025-04-14

**Authors:** Abigail Smakowski, Judith Rosmalen, Bernd Löwe, Christopher Burton, Anne Toussaint

**Affiliations:** 1 Department of Psychosomatic Medicine and Psychotherapy, University Medical Center Hamburg-Eppendorf, Hamburg, Germany; 2 Departments of Psychiatry and Internal Medicine, University of Groningen, University Medical Center Groningen, Groningen, the Netherlands; 3 Sheffield Centre for Health & Related Research, School of Medicine & Population Health, University of Sheffield, Sheffield, UK

**Keywords:** Functional somatic disorder, Functional symptoms, Persistent somatic symptoms, Persistent physical symptoms, Functional disorder, Diagnosis, General population

## Abstract

**Background:**

Persistent and troublesome physical symptoms are common and can, regardless of their cause, greatly impair patients’ quality of life. Reflecting complex brain-body interactions, they are observed across all healthcare specialties, commonly overlap across them, and receive inconsistent diagnoses. In response, the international research network EURONET-SOMA has proposed a diagnostic classification for persistent and troublesome symptoms entitled “functional somatic disorder (FSD)”. Focusing on symptom patterns across organ systems, the FSD approach aims to enhance diagnosis, treatment, and healthcare access for patients. However, further research is needed to validate its effectiveness and clinical utility. This study assessed the frequency and applicability of the FSD proposal within a population-based sample.

**Methods:**

FSD diagnostic criteria were cross-sectionally operationalised within the multi-disciplinary prospective cohort study Lifelines, conducted in the Dutch population. Kruskal–Wallis and chi-square tests with effect size estimates were used to investigate differences in the diagnostic subgroups regarding chronic diseases, functional comorbidities and psycho-behavioural features. Binary logistic regression with elastic net penalisation was used to investigate sociodemographic, psycho-behavioural and clinical factors associated with FSD.

**Results:**

Of the study population (*N* = 88,925), 58% met the diagnostic criteria for FSD. Of those meeting FSD, 31% reported a single distressing symptom, 18% had several symptoms attributable to one organ system and 52% reported multiple symptoms from various organ systems. Moderate differences between these subgroups were found for health status, neuroticism, long-term life difficulties and healthcare utilisation. Elastic net regression showed comorbid chronic musculoskeletal (OR 1.8), gastrointestinal disease (OR 1.4), neurological disease (OR 1.2), and female sex (OR 1.2) predicted FSD. Concurrent anxiety (OR 1.6), healthcare visits (OR 1.3) and long-term difficulties (OR 1.2) were associated with the presence of FSD.

**Conclusions:**

This study supports refining the FSD criteria to avoid over-inclusiveness. Current symptom severity and frequency thresholds need adjustment to better identify those needing treatment. The distinction between single and multiple symptom categories is important, and optional specifiers like comorbid chronic diagnoses and psychological factors seem valuable for predicting FSD. Despite warranting further research, the FSD classification is promising for diagnosing persistent and troublesome symptoms across medical specialties.

**Supplementary Information:**

The online version contains supplementary material available at 10.1186/s12916-025-04042-w.

## Background

Persistent and troublesome physical symptoms are observed across all healthcare specialties, commonly overlap between them, and receive inconsistent diagnoses [1]. In a recent online survey across four European countries, healthcare professionals from different medical specialties suggest the mixed terminology and varied diagnostic approaches are common barriers to treatment access for persistent physical symptoms [2]. Symptoms can persist and become troublesome, leading to impairment or disability, and can be caused by a range of biopsychosocial factors, reflecting brain-body interactions [1]. Current classification systems separate symptom-based diagnoses into somatic or mental sections; for example, somatic symptom disorder (SSD), according to the DSM-5 [3], and bodily distress disorder, according to ICD-11 [4], are mental disorder diagnoses used to classify persistent somatic symptoms that are associated with significant psychological distress. Meanwhile, functional somatic syndromes such as fibromyalgia or irritable bowel syndrome (IBS) are used within biomedical specialties (i.e. rheumatology or gastroenterology) when troublesome symptoms are associated with disability and their pathophysiological or structural basis is unknown [5, 6].

Current diagnostic criteria for these diagnoses greatly overlap, which has led to suggestions that a dimensional classification across medical specialties may be more helpful than those split by specialties [7, 8]. There has been an increasing focus within research to address diagnostic confusion and adopt a symptom-based classification that does not presuppose aetiology and allows for a biopsychosocial transdiagnostic approach [1].

### Functional somatic disorder: a recent classification proposal

This study investigates a new unifying diagnostic proposal by the European Research Network to Improve Diagnosis, Treatment, and Healthcare for Patients with Persistent Somatic Symptoms (EURONET-SOMA) to diagnose those with persistent and troublesome somatic symptoms of particular patterns in need of treatment across medical specialties. The criteria of functional somatic disorders (FSD) require the presence of “persistent and troublesome physical symptoms fitting characteristic symptom pattern(s)” [1]. FSD is designed to classify certain patterns of persistent and troublesome symptoms that cannot be better explained by another medical condition, regardless of their cause or underlying pathology [1]. Determining aetiology for persistent symptoms can be challenging as they can arise through a multitude of biopsychosocial factors that vary by individual [9]. According to the authors, the “diagnosis of FSDs should be made based on the symptoms, not on the presence or absence of specific biological or psychosocial contributors to symptoms” [1]. FSD takes a bottom-up, symptom-orientated approach considering distinct symptom subgroups determined by the affected organ systems (musculoskeletal, gastrointestinal, cardiorespiratory, genitourinary, nervous system and fatigue-related). Patients can, therefore, present with a single troublesome symptom or multiple symptoms from one or more organ systems [1]. In addition to the diagnostic criteria, FSD suggests the consideration of important contextual factors, i.e. “optional specifiers”. These include the identification of relevant chronic conditions that originate from the same organ system as the symptoms, as well as comorbid “specialty-specific” disorders (or functional somatic syndromes) such as irritable bowel syndrome (IBS) or fibromyalgia. Finally, the FSD diagnosis encourages the examination of relevant psychological features associated with the symptoms as another “optional specifier,” for example, health anxiety or avoidance behaviour [1]. FSD may have advantages over existing diagnostic classifications because it incorporates developments in aetiological research, which evidence common causes and mechanisms across various symptom-based diagnoses [10]. Although they can be diagnosed alongside a somatic disease, DSM-5 somatic symptom disorder [3] and ICD-11 bodily distress disorder [4] diagnoses are rarely used outside of psychiatry, psychosomatic medicine or clinical psychology, even though they should be applied in chronically ill patients who are significantly distressed by their symptoms. FSD is able to subsume a wider range of conditions occurring with persistent and troublesome symptoms within a neutral framework of classification systems—similar to recent chronic pain classifications in ICD-11 [4]. In terms of psychiatric disorders, such as anxiety and depression, FSD could be diagnosed in addition for patients who are especially troubled by persistent physical symptoms, such as fatigue or shortness of breath, in the context of such disorders. In terms of chronic biomedical disease, symptom burden does not always correlate with the concomitant chronic disease [11], and the longer symptoms persist, the less they can often be attributed to an originally identified biomedical cause [9, 12]. The FSD classification is intended for patients reporting persistent and troublesome physical symptoms that may not be explained by a single cause or certain pathology. It is applicable to individuals reporting symptoms from different organ systems; for instance, a person with active rheumatoid arthritis causing joint pain with swelling and fatigue may have symptoms wholly attributable to their arthritis, but as their arthritis comes under control, they develop widespread musculoskeletal pain without evidence of inflammation which has all the features of fibromyalgia. The classification is also applicable to those whose symptoms are limited to a single organ system or involve a single troublesome symptom only, such as chronic headache. Clinical application of the classification requires that symptoms align with FSD’s characteristic patterns after considering other biomedical and psychiatric differential diagnoses. FSD aims to offer a neutral disease classification, avoiding assumptions about somatic or mental origins, which is particularly useful for bridging gaps between medical specialties. With the option of assigning it alongside other medical conditions, FSD could enable the development of diagnostic referral pathways across medical specialties and facilitate tailored, personalised treatments informed by new evidence from different medical fields.

### Current evidence for FSD and symptom-based diagnoses

Although the FSD classification was derived from a synthesis of research results, it currently has only limited empirical evidence. Diagnostic concepts must be assessed for their ability to identify patients in need who will benefit from treatment. New diagnostic proposals need to be evaluated in context to determine their clinical utility and validity in patient groups of interest. FSD must prove its reliability, validity and diagnostic accuracy in comparison to other existing diagnoses. So far, FSD has only been evaluated in one study [13], which supports the concept in a sample from the German general population, confirming distinct symptom patterns that appear in one or multiple organ systems. However, the authors call for refined definitions for the severity threshold of symptoms within the FSD criteria; they suggest two severity grades according to high and low symptom burden. Overall, repeated testing of the new diagnostic proposal in further population-based and clinical samples is required; the frequencies of the defined specifier disorders must be determined, and different symptom severity thresholds should be tested.

### Aims and objectives

This study provides insights into the applicability of the FSD diagnosis in a large cohort of the Dutch general population and its potential utility in medical care in the future. First, we investigated the frequency of the FSD diagnosis and its associations with sociodemographic and clinical characteristics. Next, we compared the diagnostic subcategories of FSD (single-symptom, single-system, and multi-system subgroups) with non-cases in relation to clinical features and relevant outcomes according to the FSD criteria. Finally, we investigated relevant predictors and associates of FSD.

## Methods

### Study design, setting and data sources

This study is part of the innovative training network ETUDE (Encompassing Training in fUnctional Disorders across Europe; https://etude-itn.eu/), a network that aims to improve the understanding of mechanisms, diagnosis, treatment and stigmatisation of functional disorders [14]. Pre-registration for the study can be accessed via the Open Science Framework (https://doi.org/10.17605/OSF.IO/JRB6K).

The study utilised data from Lifelines [15, 16], a multi-disciplinary prospective population-based cohort study examining in a three-generation design the health and health-related behaviours of a total of 167,729 persons living in the North of the Netherlands. It employs a broad range of investigative procedures in assessing the biomedical, socio-demographic, behavioural, physical and psychological factors which contribute to the health and disease of the general population, with a special focus on multi-morbidity and complex genetics. The sample is considered representative of the Dutch population [17]. An in-depth breakdown of the Lifelines study design is available [18]. Lifelines is approved by the UMCG Medical ethical committee (2007/152).

Lifelines questionnaires were administered on-site every five years and online to participants every 1.5 years. For this study, we utilised data from the subset of cohort members (*n* = 88,952) who completed a questionnaire at the second cohort assessment (between 2014 and 2017) and some additional data from the first cohort assessment as potential predictors for FSD (2007–2013). Participants were excluded if they did not provide basic demographic data.

### FSD case definition

**Fig. 1:**
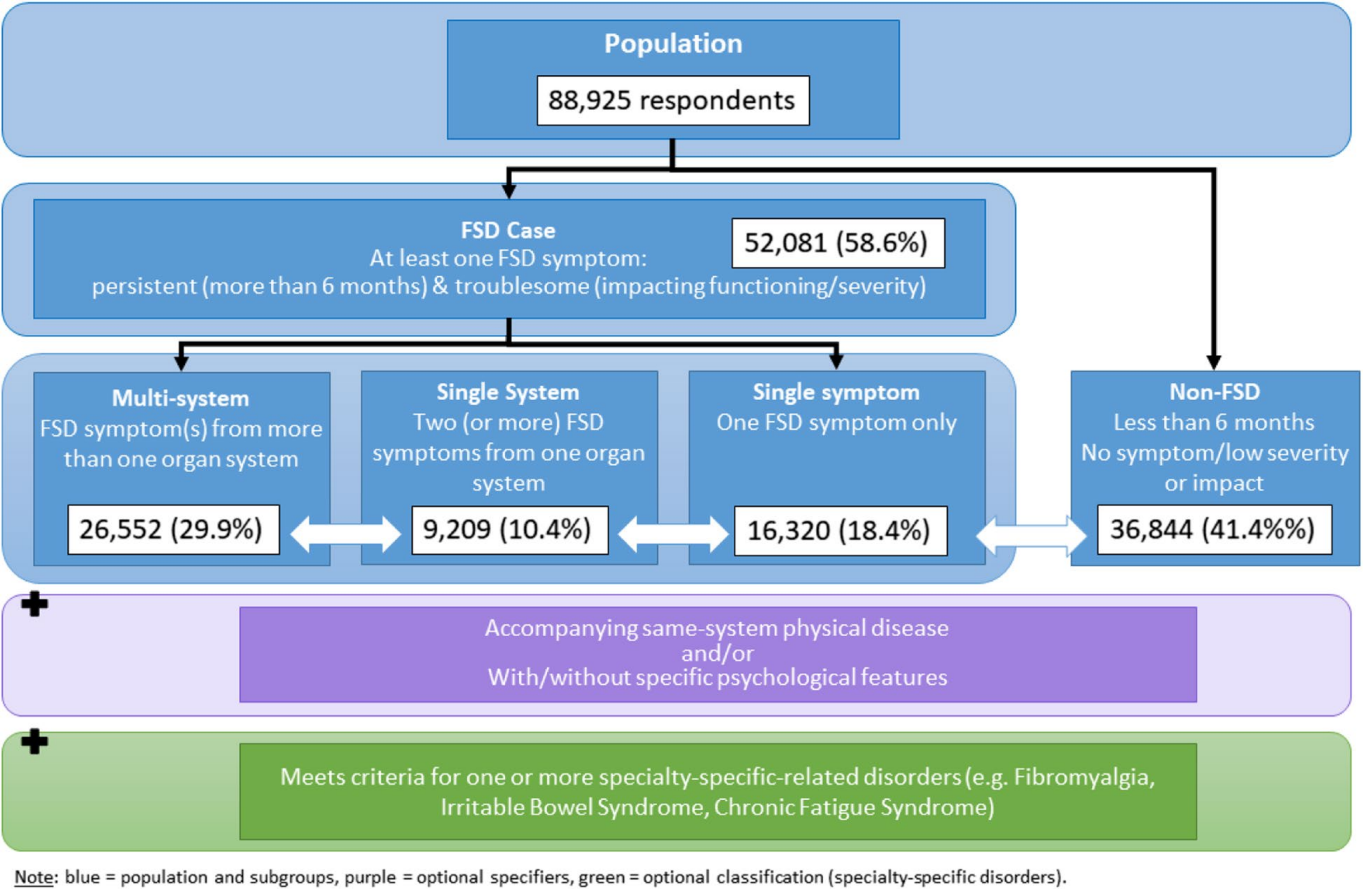
Adapted figure of FSD classification for the purposes of this study, based on Burton et al. [1]

This study operationalised the diagnostic conceptualisation of FSD as proposed by Burton et al. (2020) to identify people in the population that would meet the FSD criteria, Fig. 1 presents a diagram of the classification of FSD, adapted for this study. FSD requires the presence of at least one “troublesome persistent symptom” [1]. Using validated measures of somatic symptom severity (BDS Checklist [19] and PHQ-15 [20]) as a guide, we established a list of relevant symptoms. Then, we cross-referenced them with self-reported symptoms provided in Lifelines. The FSD framework recommends 3 months to define persistence; however, since Lifelines uses 6 months for most symptom measures, we decided upon a cut-off of 6 months for practicality. Therefore, “persistent symptoms” were defined as lasting at least 6 months, and “troublesome symptoms” as moderately to severely impacting a person’s life based on the information in the Likert scales of the respective measuring instruments. Specifically, symptoms, their duration, and impact were determined using information provided by the following validated measures (Supplementary File 4 for all symptoms and source variables):

- The Widespread Pain Index (WPI) was developed as part of the Fibromyalgia Survey Questionnaire (FSQ) and used to diagnose pain-related disorders such as fibromyalgia [21]. In this study, for FSD symptoms, the WPI indicated the location of pain across the body combined with duration measures (“I have had my musculoskeletal pain complaints for about: …”) for which an answer of 6 months or longer was required. Impact due to symptoms was measured via the question, “to what extent did your musculoskeletal pain hamper your normal activities (both work outside the home and household chores) in the past six months?” to which an answer of “quite a bit”, “a lot”, or “very much” was required.
- ROME-III [6]—for the assessment of conditions such as IBS [22]—was used to investigate frequent loose bowel movements, abdominal pains, feeling bloated, hard stools, constipation, burning sensation in upper stomach, unpleasant bloating after meals and inability to complete meals. The impact of FSD symptoms required respondents to experience the symptom for longer than 6 months “often”, “most of the time”, or “always” on 3 days per month, or hampering normal activities “quite a bit”, “a lot”, or “very much”, depending on the symptom.
- CDC symptom inventory [23] by the US Centers for Disease Control and Prevention (CDC), which collects information on fatigue and related symptoms, was used to investigate symptoms of muscle pain and joint pain. FSD symptoms were required to occur “a few times a week” or “everyday” for longer than 6 months.
- The Checklist Individual Strength (CIS) questionnaire [24] was used to determine troublesome fatigue. CIS responses were calculated to indicate severity using the 35-point recommended cut-off, combined with a variable that indicated the fatigue had persisted for over 6 months.
- The Symptom Checklist (SCL-90) somatization scale (SCL-SOM) was used to investigate the severity of difficulty breathing, hot and cold spells, nausea, localised weakness, numbness (or tingling sensation) and dizziness [25]. Since the SCL-SOM does not measure duration, persistence was defined as reporting moderate to severely impacting symptoms at two time points, which are, on average, 16 months (± 10 months) apart, thus indicating symptom persistence.

### FSD diagnostic subcategories: single symptom, single system, and multi system

Symptoms were categorised by associated organ systems, defined by those recommended within the FSD framework [1] and the availability of appropriate symptoms in the Lifelines data set, i.e. cardiorespiratory, gastrointestinal, musculoskeletal, and neurological organ systems. According to the framework, FSD cases can be sub-classified according to those who report only one persistent troublesome symptom, those who report multiple symptoms from the same organ system, and those who report multiple persistent troublesome symptoms from more than one organ system. The total number of persistent troublesome symptoms and the total number of organ systems involved were calculated for each participant. A list of all symptoms and their assigned organ system is found in Supplementary File 3.

### Optional specifiers

#### Accompanying same-system physical disease

We collected all self-reported biomedical diseases reported by Lifelines participants at any measurement point and matched them to a corresponding organ system following the logic of the symptom clusters (e.g. gastrointestinal). From here, the number of FSD cases reporting a same-system physical disease was calculated. Supplementary File 2 provides a list of all chronic diseases included in this study. Conditions such as “cancer” that were not clearly attributable to one of the defined organ systems were excluded from analyses.

#### Specific psychological features

As an optional specifier, the FSD framework incorporates psychological or behavioural features that “cause distress, beyond (what is)… caused by the symptoms themselves” [1]. In this study, we utilised the self-administered MINI interview [26, 27] to assess how many FSD cases also fulfilled the diagnostic criteria of a concurrent depression or anxiety disorder. In addition to this, we included psychological factors known to be associated with symptom-based diagnoses [28]. These factors were measured by self-report questionnaires and included the Childhood Trauma Questionnaire (CTQ) [29, 30], Threatening Life Events for the last 6 months (LTE) [31], the Loneliness scale [32] and the NEO-neuroticism questionnaire [33].

### Specialty-specific disorders

The FSD diagnosis recommends further specification by recording dual diagnoses of functional somatic syndromes. Three of the most common are assessed by a combination of self-report measures in Lifelines. Chronic fatigue syndrome (CFS) was measured according to the Centers for Disease Control and Prevention [34]. Fibromyalgia was measured according to the American College of Rheumatology [5]. IBS was measured by ROME-III criteria [22], with adjustments to account for the ROME-VI [35, 36]. More details on the operationalisation process of these criteria within Lifelines are available [36].

### Additional variables

Socio-demographic variables such as age, sex and ethnicity were analysed to describe the study sample and compare the diagnostic subgroups. Healthcare visits, measured by the number of different types of healthcare providers visited in the past year, and physical functioning, measured by the EQ-5D-5L [37], were included as relevant clinical outcomes.

### Statistical comparisons

Frequency rates and socio-demographic and clinical characteristics of those who met the criteria for FSD and the subgroups are described. Frequencies of those with accompanying comorbidities, including a “same-system physical disease” and “specialty-specific disorder,” were reported.

A series of Kruskal–Wallis tests were conducted to compare differences in continuous variables between the subgroups of FSD (single-symptom, single-system, multi-system) and non-cases. Pairwise comparisons were performed using Dunn’s (1964) procedure with a Bonferroni correction for multiple comparisons; therefore, statistical significance was accepted at the *p* < 0.0083 level. Chi-square tests compared categorical social-demographic variables and comorbid conditions between subgroups. In large datasets, hypothesis testing often shows significant between-group differences because they are based on power; therefore, we provided effect sizes to assess whether differences are meaningful: Cramer’s V for categorical data (small > 0.1, medium > 0.3, large > 0.5) and Epsilon Squared for continuous data (0.00–0.01 negligible < weak 0.04 < moderate 0.16 < relatively strong 0.36). Further, post hoc tests were conducted to ensure that the conclusions about group differences are statistically robust and reliable.

Binary logistic regression with elastic net regularisation was conducted to predict group membership, i.e. meeting the FSD classification compared to not meeting the criteria [38]. Elastic net regression was applied in this study to prevent the model over-fitting factors in large samples. Elastic net has the combined benefits of Ridge regression (ability to handle multicollinearity) and Lasso regression (ability to handle many predictors by variable selection). The penalisation procedure involved randomly partitioning data into a test (20%) and training set (80%), then, optimal regularisation parameter alpha and lambda with the lowest mean square error were obtained by tenfold cross-validation [39, 40]. Cross-validation is a machine learning procedure, involving dividing the available data into subsections (or folds), using one as a validation set, and training the model on the remaining folds. This method is repeated using different folds as the validation set, and then the results are averaged to produce a more robust estimate of the model’s performance. The area under the receiver operating curve (AUC) was used to assess the accuracy of predicted classifications of FSD cases and non-cases where 0.7 to 0.8 is considered acceptable [41].

Subgroup comparisons were conducted using IBM statistics version 29.0.1. Regression analysis was run with R project software (version 4.4.0). Supplementary File 1 details all variables used in this study and the respective statistics.

## Results

### Frequency of the FSD diagnosis and sociodemographic and psycho-behavioural characteristics of participants meeting the classification

Of the total Lifelines cohort, 88,952 participants were included in this study. The median age of the total sample was 50 (range 18–95). There were more females (*n* = 52,539, 59%), and most were white European (*n* = 80,013, 90%).

As shown in Table 1 and Fig. 1, a large number of the Lifelines population met the criteria for FSD (*n* = 52,081, 58%). The majority of the FSD cases were females (*n* = 33,877, 65%), a higher proportion than in non-cases (*n* = 18,662, 51%). More FSD cases reported depression (*n* = 2370, 5% versus non-cases *n* = 269, 1%) or a concurrent diagnosis of anxiety (*n* = 4576, 9% versus non-cases *n* = 709, 2%). FSD cases reported more chronic diseases from all organ systems than non-cases. Cardiorespiratory conditions were reported with the highest frequencies in the population (*n* = 21,422, 41% versus non-cases *n* = 12,119, 33%), followed by neurological/other (*n* = 13,474, 26% versus non-cases *n* = 5867, 16%), musculoskeletal (*n* = 8013, 15% versus non-cases *n* = 2572, 7%) and gastrointestinal conditions (*n* = 4297, 8% versus non-cases *n* = 1492, 4%). Thirteen per cent (*n* = 6501) of FSD cases met criteria for a concurrent specialty-specific diagnosis (CFS, FM, or IBS); a small proportion met two (*n* = 1943, 4%) and a very small proportion met all three diagnostic groups (*n* = 463, 1%).

**Table 1:** Frequencies and socio-demographic characteristics of FSD-cases versus non-cases

**Characteristic**		**FSD-cases *N* = 52,081 (58.6%)**	**Non-cases *N* = 36,846 (41.4%)**	***p* value**
Mean age (SD)		50.30 (12.56)	51.03 (12.93)	*p* < 0.001
Sex (%)	Male	18,204 (35.0%)	18,184 (49.4%)	*p* < 0.001
	Female	33,877 (65.0%)	18,662 (50.6%)	
Ethnicity	White/eastern and western Europe	46,710 (89.6%)	33,303 (98.7%)	*p* = 0.224
	Other	680 (1.4%)	450 (1.3%)	
Current relationship duration in years (SD)		24.58 (13.98)	25.73 (13.85)	*p* < 0.001
Mean of people living in household (SD)		2.75 (1.28)	2.81 (1.37)	*p* < 0.001
Education	Low	13,224 (25.5%)	9591 (26.0%)	*p* = 0.19
	Medium	16,256 (31.2%)	11,208 (30.4%)	
	High	14,124 (27.1%)	9895 (26.9%)	
Mean number of paid working hours (SD)		30.26 (12.52)	32.75 (12.79)	*p* < 0.001
Number with a concurrent psychiatric diagnosis (%)	Depression	2370 (4.6%)	269 (0.7%)	*p* < 0.001
	Anxiety	4576 (8.8%)	709 (1.9%)	*p* < 0.001
Number with a chronic disease from a particular body system (%)	Cardiorespiratory	21,422 (41.1%)	12,119 (32.9%)	*p* < 0.001
	Musculoskeletal	8013 (15.4%)	2572 (7.0%)	*p* < 0.001
	Gastrointestinal	4297 (8.3%)	1492 (4.0%)	*p* < 0.001
	Neurological/other	13,474 (25.9%)	5867 (15.9%)	*p* < 0.001
Number meeting criteria for a concurrent Functional Syndrome (%)				
	Fibromyalgia	5220 (10.0%)	100 (0.3%)	*p* < 0.001
	Irritable bowel syndrome	4894 (9.4%)	< 10 (0.0%)	*p* < 0.001
	Chronic fatigue syndrome/ME	2790 (5.4%)	< 10 (0.0%)	*p* < 0.001
Number meeting one or more functional syndrome criteria (%)	No syndrome	37,575 (72.1%)	33,373 (90.6%)	*p* < 0.001
	One syndrome	6501 (12.5%)	103 (0.3%)	*p* < 0.001
	Two syndromes	1943 (3.7%)	-	
	All three syndromes	463 (0.9%)	-	
Number of systems involved for FSD cases	Symptom(s) from one system	25,534 (49.0%)	-	
	Symptom(s) from two systems	17,046 (32.7%)	-	
	Symptom(s) from three systems	8145 (15.6%)	-	
	Symptom(s) from all systems	1356 (2.6%)	-	

Note: all data are valid frequencies and valid per cent

The five most frequently reported FSD symptoms in the total sample were fatigue (*n* = 21,054, 24%), feeling bloated (*n* = 21,372, 24%), unrefreshing sleep (*n* = 15,757, 18%), abdominal pain (*n* = 14,539, 16%) and joint pain (*n* = 13,221, 15%). Most FSD cases experienced symptoms from one organ system only (*n* = 25,534, 49%), compared to two (*n* = 17,046, 33%), three (*n* = 8145, 16%), or all four systems (*n* = 1356, 3%). Supplementary Files 4 and 5 provide frequency data for FSD symptoms.

### Comparing the subgroups of FSD classification and non-cases

Complete data for subgroup comparison analysis was available for non-cases (*n* = 36,846), single-symptom (*n* = 16,321), single-system (*n* = 9213) and multi-system (*n* = 26,547) FSD cases. Table 2 provides frequency data for socio-demographic and other relevant characteristics by FSD subgroups. Supplementary File 6 provides a visual flow of the population divided by subgroups of FSD and the relative proportions of relevant comorbidities. Kruskal–Wallis comparisons found moderate effect sizes for health status, neuroticism, and long-term difficulties (Table 3). Post hoc tests indicated the largest differences and poorer outcomes for multi-system cases compared to non-cases and single-symptom cases. Small effect sizes were observed between subgroups for threatening experiences, hours working per week, loneliness, childhood trauma and the number of healthcare practitioners visited. Post hoc patterns showed the biggest differences were between multi-system and non-cases and single symptom cases and those meeting a system classification versus those with a single symptom (Table 3).

**Table 2:** Characteristics and frequencies of FSD cases split by subgroup

Characteristic	Subcategory	Non-cases	Single symptom	Single system	Multi-system
Frequency in total population (% of FSD cases)		36,846 (–)	16,321 (31.3%)	9213 (17.7%)	26,547 (51.9%)
Mean age (SD)		51.03 (12.93)	50.17 (12.70)	49.03 (12.71)	50.82 (12.39)
Sex (%)	Male	18,184 (49.4%)	6805 (41.7%)	3226 (35.0%)	8173 (30.8%)
	Female	18,662 (50.6%)	9516 (58.3%)	3226 (35.0%)	18,374 (69.2%)
Ethnicity	White/eastern and western Europe	33,303 (90.4%)	14,823 (90.8%)	8259 (89.6%)	23,628 (89.6%)
	White/Mediterranean or Arabic	73 (0.2%)	38 (0.2%)	18 (0.2%)	83 (0.3%)
	Black	37 (0.1%)	23 (0.1%)	10 (0.1%)	34 (0.1%)
	Asian	164 (0.4%)	55 (0.3%)	32 (0.3%)	103 (0.4%)
	Other	372 (1.0%)	78 (0.5%)	50 (0.5%)	156 (0.6%)
Current relationship duration in years (SD)		25.73 (13.85)	24.79 (13.85)	23.63 (13.81)	24.77 (14.11)
Number of people living in household (SD-maybe range)		2.81 (1.37)	2.81 (1.26)	2.82 (1.36)	2.68 (1.26)
Education	Low	9591 (26.0%)	3856 (23.6%)	2107 (22.9%)	7261 (27.4%)
	Medium	11,208 (30.4%)	4980 (30.5%)	2890 (31.4%)	2890 (31.4%)
	High	9895 (26.9%)	4815 (29.5%)	2707 (29.4%)	2707 (29.4%)
Number of paid working hours (SD)		32.75 (12.79)	31.49 (12.55)	30.56 (12.22)	29.31 (12.54)
Median number of healthcare providers visited in the past year (IQR)		2 (2)	2 (1)	3 (2)	3 (2)
Number with a concurrent psychiatric diagnosis (%)	Depression	269 (0.7%)	260 (1.6%)	314 (3.4%)	1796 (6.8%)
	Anxiety	709 (1.9%)	626 (3.8%)	747 (8.1%)	3203 (12.1%)
Number with same-system comorbidity according to FSD criteria	Cardiorespiratory	-	36 (0.2%)	< 10 (0.0%)	1263 (4.8%)
	Musculoskeletal	-	722 (4.4%)	428 (4.6%)	4758 (17.9%)
	Gastrointestinal	-	454 (2.8%)	397 (4.3%)	2252 (8.5%)
	Neurological/other	-	1431 (8.8%)	1023 (11.1%)	7099 (26.7%)
Number meeting criteria for a concurrent Functional Syndrome (%)	Fibromyalgia	100 (0.3%)	152 (0.9%)	241 (2.6%)	4827 (18.2%)
	Irritable bowel syndrome	< 10 (0.0%)	177 (1.1%)	928 (10.1%)	3789 (14.3%)
	Chronic fatigue syndrome/ME	< 10 (0.0%)	19 (0.1%)	43 (0.5%)	2728 (10.3%)
Median total number of symptoms meeting FSD criteria (IQR)		-	1 (0)	2 (1)	4 (3)

Note: these frequencies will not match those used in the statistical subgroup comparison analyses as these required complete data. For more detailed information on specific variables please see Supplementary File 1

**Table 3:** Continuous variable results of FSD subgroup comparisons

Variable	Measure	Non-case	Single-symptom	Single-system	Multi-system	Hypothesis test	Effect size	Post hoc comparison
	Count n	36,846	16,321	9213	26,547			
**Age**								
	Mean SD	51.0 (12.9)	50.2 (12.7)	49.0 (12.7)	50.8 (12.4)			
	Median IQR	51 (17)^c,e^	50 (16)^a,d,e^	49 (16)^a,b,c^	51 (16)^b,d^	(*H* (3) = 213.1, *p* < 0.001)	0.0024	^a^(7.4) ^b^(− 12.4) ^c^(13.5) ^d^(− 5.5) ^e^(6.6), (*p* < .001)
**Health Status EQ-5D-5L**								
	Mean SD	84.1 (24.7)	81.1 (23.9)	78.8 (29.7)	72.6 (25.9)			
	Median IQR	89 (15)^c,e,f^	85 (11)^b,d,f^	80 (15)^a,d,e^	78 (16)^a,b,c^	(*H* (3) = 10,534.0, *p* < 0.001)	0.118	^a^(30.1) ^b^(57.2) ^c^(101.4) ^d^(15.7) ^e^(38.8) ^f^(26.4), (*p* < .001)
**Number in household**								
	Mean SD	2.81 (1.37)	2.81 (1.26)	2.82 (1.36)	2.68 (1.26)			
	Median IQR	2 (2)^a^	2 (2)^b^	2 (2)^c^	2 (2)^a,b,c^	(*H* (3) = 194.3, *p* < 0.001)	0.00218	^a^(12.4) ^b^(10.2) ^c^(9.1), (*p* < .001)
**Long-term difficulties LDI**								
	Mean SD	1.32 (1.69)	1.85 (1.98)	2.32 (2.27)	2.81 (2.55)			
	Median IQR	1 (2)^a,b,c^	1 (3)^a,d,e^	2 (2)^b,d,f^	2 (3)^c,e,f^	(*H* (3) = 7044.9, *p* < 0.001)	0.0792	^a^(− 30.0) ^b^(− 41.2) ^c^(− 81.9) ^d^(− 15.3) ^e^(− 38.1) ^f^(− 14.8), (*p* < .001)
**List of threatening experiences LTE**								
	Mean SD	0.64 (0.95)	0.72 (0.99)	0.80 (1.07)	0.95 (1.18)			
	Median IQR	0 (1)^a,b,c^	0 (1)^a,d,e^	0 (1)^b,d,f^	1 (2)^c,e,f^	(*H* (3) = 1126.0, *p* < 0.001)	0.0127	^a^(− 8.2) ^b^(− 12.4) ^c^(− 33.1) ^d^(− 5.2) ^e^(− 19.1) ^f^(− 10.1), (*p* < .001)
**Neuroticism NEO**								
	Mean SD	25.1 (6.69)	26.4 (7.02)	27.8 (7.43)	29.3 (7.92)			
	Median IQR	24 (7.8)^a,b,c^	26.4 (9.6)^a,d,e^	27 (11)^b,d,f^	28.8 (10)^c,e,f^	(*H* (3) = 4084.2, *p* < 0.001)	0.0459	^a^(− 18.4) ^b^(− 29.3) ^c^(− 62.5) ^d^(− 12.9) ^e^(− 33.5) ^f^(13.7), (*p* < .001)
**Relationship duration**								
	Mean SD	25.7 (13.8)	24.8 (13.8)	23.6 (13.8)	24.8 (14.1)			
	Median IQR	25 (21)^c,d,e^	25 (21)^b,e^	24 (22)^a,b,c^	25 (22)^a,d,e^	(*H* (3) = 155.1, *p* < 0.001)	0.00174	^a^(− 6.1) ^b^(5.8) ^c^(11.5) ^d^(7.4) ^e^(6.3), (*p* < .001)
**Hours working per week**								
	Mean SD	32.7 (12.8)	31.5 (12.5)	30.6 (12.2)	29.3 (12.5)			
	Median IQR	36 (16)^c,e,f^	32 (16)^b,d,f^	32 (16)^a,d,e^	30 (18)^a,b,c^	(*H* (3) = 918.32, *p* < 0.001)	0.0103	^a^(6.8) ^b^(15.5) ^c^(29.7) ^d^(5.6) ^e^(14.0) ^f^(9.6), (*p* < .001)
**Loneliness**								
	Mean SD	0.63 (1.11)	0.81 (1.26)	0.97 (1.39)	1.24 (1.52)			
	Median IQR	0 (1)^a,b,c^	0 (1)^a,d,e^	0 (2)^b,d,f^	1 (2)^c,e,f^	(*H* (3) = 1985.1, *p* < 0.001)	0.0223	^a^(− 11.5) ^b^(− 16.8) ^c^(− 44.0) ^d^(− 6.9) ^e^(− 24.8) ^f^(− 12.8), (*p* < .001)
**Childhood trauma CTQ**								
	Mean SD	33.4 (8.00)	34.2 (8.73)	35.2 (9.73)	37.0 (11.4)			
	Median IQR	32 (8)^a,b,c^	32 (9)^a,d,e^	33 (10)^b,d,f^	34 (12)^c,e,f^	(*H* (3) = 1119.2, *p* < 0.001)	0.0126	^a^(− 6.8) ^b^(− 12.2) ^c^(− 32.8) ^d^(− 6.1) ^e^(− 20.2) ^f^(− 9.9), (*p* < .001)
**Number of health practitioners visited**								
	Mean SD	2.14 (1.20)	2.46 (1.27)	2.73 (1.34)	3.17 (1.50)			
	Median IQR	2 (2)^a,b,c^	2 (1)^a,d,e^	3 (2)^b,d,f^	3 (2)^c,e,f^	(*H* (3) = 7486.8, *p* < 0.001)	0.0842	^a^(− 25.0) ^b^(− 35.7) ^c^(− 85.4) ^d^(− 13.8) ^e^(− 45.5) ^f^(− 22.5), (*p* < .001)

Note: *H* Kruskal–Wallis test, effect size test for continuous variables = epsilon squared (.00–.01 negligible < weak .04 < moderate .16 < relatively strong .36)

Secondly, a series of chi-squared tests were conducted between subgroups of FSD, non-cases, and relevant categorical variables (see Table 4). There was a statistically significant difference between all variables, which was unsurprising given the large sample; however, differences varied in effect size. Ethnicity and education showed negligible effect sizes for differences between groups. In contrast, sex, diagnosis of anxiety, depression, coexisting chronic cardiorespiratory, gastrointestinal, musculoskeletal, and neurological disease, and concurrent CFS or IBS showed small effects. Moderate differences between subgroups were found for those with co-occurring fibromyalgia. Single-symptom and single-symptom subgroups consistently displayed the lowest proportions of co-occurring functional conditions across all subgroups.

**Table 4:** Categorical variable results of FSD subgroup comparisons

Variable	Measure	Non-case	Single-symptom	Single-system	Multi-system	Hypothesis test	Effect size
	Count *n*	36,846	16,321	9213	26,547		
Sex							
	Female	18,662 (35.5%)	9516 (18.1%)	5987 (11.4%)	18,374 (35.0%)	*χ*^2^ (3) = 2347.9, *p* < .001	0.162
	Male	18,184 (50.0%)	6805 (18.7%)	3226 (8.9%)	8173 (22.5%)		
Ethnicity							
	Not White European	450 (39.8%)	194 (17.2%)	110 (9.7%)	379 (33.3%)	*χ*^2^ (3) = 7.6, *p* = .054	0.054
	White European	33,303 (41.6%)	14,823 (18.5%)	8259 (10.3%)	23,628 (29.5%)		
Education level							
	Low	9591 (42.0%)	3856 (16.9%)	2107 (9.2%)	7261 (31.8%)	*χ*^2^ (6) = 195.4, *p* < .001	0.036
	Medium	11,208 (40.8%)	4980 (18.1%)	2890 (10.5%)	8386 (30.5%)		
	High	9895 (41.2%)	4815 (20.0%)	2707 (11.3%)	6602 (27.5%)		
Concurrent anxiety disorder							
	No anxiety diagnosis	26,376 (43.7%)	11,553 (19.1%)	6137 (10.2%)	16,346 (27.1%)	*χ*^2^ (3) = 3128.6, *p* < .001	0.218
	Anxiety diagnosis	709 (13.4%)	626 (11.8%)	747 (14.1%)	3203 (60.6%)		
Concurrent depression disorder							
	No depression diagnosis	26,816 (42.5%)	11,919 (18.9%)	6570 (10.4%)	17,753 (28.2%)	*χ*^2^ (3) = 2114.9, *p* < .001	0.179
	Depression diagnosis	269 (10.2%)	260 (9.9%)	314 (11.9%)	1796 (68.1%)		
Concurrent same-system chronic disease							
	No concurrent disease	36,846 (51.0%)	13,678 (18.9%)	7365 (10.2%)	14,379 (19.9%)	*χ*^2^ (3) = 21,382.0, *p* < .001	0.49
	At least one chronic disease	0 (0.0%)	2643 (15.9%)	1848 (11.1%)	12,168 (73.0%)		
Cardiorespiratory chronic disease							
	No	24,727 (44.6%)	10,380 (18.7%)	5798 (10.5%)	14,481 (26.1%)	*χ*^2^ (3) = 1055.0, *p* < .001	0.109
	Yes	12,119 (36.1%)	5941 (17.7%)	3415 (10.2%)	12,066 (36.0%)		
Gastrointestinal chronic disease							
	No	35,354 (42.5%)	15,423 (18.6%)	8549 (10.3%)	23,812 (28.6%)	*χ*^2^ (3) = 1028.6, *p* < .001	0.108
	Yes	1492 (25.8%)	898 (15.5%)	664 (11.5%)	2735 (47.2%)		
Musculoskeletal chronic disease							
	No	34,274 (43.7%)	14,576 (18.6%)	8251 (10.5%)	21,241 (27.1%)	*χ*^2^ (3) = 2547.6, *p* < .001	0.169
	Yes	2572 (24.3%)	1745 (16.5%)	962 (9.1%)	5306 (50.1%)		
Neurological/other chronic disease							
	No	30,979 (44.5%)	12,940 (18.6%)	6939 (10.0%)	18,728 (26.9%)	*χ*^2^ (3) = 1717.6, *p* < .001	0.139
	Yes	5867 (30.3%)	3381 (17.5%)	2274 (11.8%)	7819 (40.4%)		
Concurrent FD							
	Did not meet FD criteria	36,740 (46.6%)	15,974 (20.2%)	8029 (10.2%)	18,167 (23.0%)	*χ*^2^ (3) = 1677.5, *p* < .001	0.434
	One or more FD criteria	106 (1.1%)	347 (3.5%)	1184 (11.8%)	8380 (83.7%)		
Met CDC criteria for CFS							
	No	36,664 (42.9%)	16,243 (19.0%)	9115 (10.7%)	23,450 (27.4%)	*χ*^2^ (3) = 6400.6, *p* < .001	0.269
	Yes	< 10 (< x%)	19 (0.7%)	43 (1.5%)	2728 (97.7%)		
Met ROME-III criteria for IBS							
	No	36,746 (43.9%)	16,101 (19.2%)	8242 (9.9%)	22,579 (27.0%)	*χ*^2^ (3) = 7074.5, *p* < .001	0.283
	Yes	< 10 (< x%)	177 (3.6%)	928 (19.0%)	3789 (77.4%)		
Met WPI criteria for fibromyalgia							
	No	33,548 (44.6%)	14,694 (19.5%)	8095 (10.7%)	18,967 (25.2%)	*χ*^2^ (3) = 10,335.9, *p* < .001	0.358
	Yes	100 (1.9%)	152 (2.9%)	241 (4.5%)	4827 (90.7%)		

Note: Chi-square test, effect size = Cramer’s V (small > 0.1, medium > 0.3, large > 0.5). Ethnicity variables was converted into binary format due to frequency distribution. *FD*, functional disorder (CFS/fibromyalgia/IBS)

### Predictors and associates of FSD diagnosis

Elastic net regression was applied to participants with complete data (*n* = 20,262) to identify the variables that best predict a diagnosis of FSD in the population. After cross-validation, the optimal regularisation parameter was found using alpha = 1.0. With lambda set to 0.008 (alpha = 1), the model achieved an AUC of 0.67, close to the recommended range of 0.7 to 0.8. [41]. Table 5 shows the coefficients and odds ratios contributing to the model. Odds ratios (ORs) above 1.0 indicate a greater likelihood of a FSD diagnosis. A chronic condition from the musculoskeletal organ system resulted in a higher likelihood of meeting the FSD criteria (OR 1.8), followed by gastrointestinal chronic conditions (OR 1.4). The neurological chronic condition cluster was a weaker predictor of FSD (OR 1.2). In terms of psychological associates of FSD, a concurrent diagnosis of anxiety indicated someone was more likely also to meet the criteria for FSD (OR 1.6); so did long-term difficulties (OR 1.2). A higher degree of loneliness only indicated a small increase in the odds of FSD (OR 1.1). Regarding demographics, male sex was predictive of being a non-case (OR 0.8). In terms of healthcare utilisation, visiting more healthcare practitioners was associated with an increased likelihood of FSD (OR 1.3). Other variables had little to no contribution according to the model coefficients, such as health status according to EQ-5D, work duration, ethnicity, neuroticism, co-occurring cardiorespiratory condition, relationship duration, depression and childhood trauma.

**Table 5:** Predictors and associates of FSD binary logistic regression with elastic net penalisation

Model features	Penalised coefficients (λ.1se, alpha = 1)	Odds ratio
Musculoskeletal chronic condition	0.58	1.78
Anxiety (concurrent)	0.45	1.57
Gastrointestinal chronic condition	0.34	1.40
Healthcare practitioners visited	0.23	1.25
Neurological chronic condition	0.18	1.19
Long term difficulties (LDI)	0.19	1.21
Male sex	− 0.23	0.79
Loneliness	0.06	1.06
Neuroticism (NEO)	0.02	1.02
Childhood trauma (CTQ)	0.01	1.00
Working hours in a week	− 0.00	1.00
Health status (EQ-5D)	− 0.00	1.00
Cardiorespiratory chronic condition	.	.
Number living in house	.	.
Education—middle	.	.
Threatening experiences (LTE)	.	.
Age	.	.
Depression (concurrent)	.	.
White ethnicity	.	.
Relationship duration (years)	.	.
Education—high	.	.

Note: Area under the curve for the model = 67%. Features with low coefficients contribute less to the final model. The absolute value of each feature coefficient is roughly proportional to its importance. Odds ratios above 1 predict FSD. The coefficients represent the relationship between the predictor and response variables. “.” = indicated variable does not contribute to the model. Analyses are designed to “shrink” unimportant variables to zero and select the most important to prevent overfitting and improve model accuracy

## Discussion

### Frequencies of FSD and characteristics of people meeting FSD criteria in comparison to existing diagnostic classifications

Functional somatic disorder (FSD) by the EURONET-SOMA group is a diagnostic framework proposal for classifying persistent and troublesome symptoms lasting at least three months. It categorises symptoms based on affected organ systems and aims to unify diagnosis across specialties while considering biomedical and psychological factors to improve patient care. Our study’s first major finding was that 58% of the Lifelines population reported at least one somatic symptom persisting for 6 months with moderate severity or impairment. We understand that not all of these symptoms may have disease value and individuals may not necessarily seek treatment for these symptoms. However, the number seems comparable to figures found in primary care data registries in the Netherlands, where 58% of 28,590 patients who contacted their general practitioner reported at least one symptom diagnosis in the last year [42]. Prevalence rates of diagnoses based on persistent and troublesome somatic symptoms that require clear exclusion of pathophysiology to explain the symptoms such as the former DSM-IV somatoform disorders are much lower, and range between 11 and 21% [43–45]. The same is true for the prevalence of functional syndromes in the general population, i.e. prevalence for irritable bowel syndrome according to ROME-VI is 4% [46], for fibromyalgia it is reported to be 2% [47] and 1% for CFS [48]. It is of course possible that the purely symptomatic FSD criteria also identify people with chronic physical disease that may explain the symptoms; in our population-based data set, we were not able to consider differential diagnoses before application of the FSD criteria. However, our prevalence data (Table 2) indicate that self-reported biomedical diagnoses affecting the same organ system as the FSD symptoms were reported by only 0.1 to 11% of individuals with single system FSD and for any of the same organ systems in 4.8 to 26.7% of individuals with multi-system FSD.

Given that our study utilised a representative sample of the Dutch general population, it is unlikely that the prevalence of FSD in our sample is substantially over- or underestimated. Any potential variation may instead stem from methodological considerations. The only other study assessing the FSD proposal to date found a lower frequency of persistent and troublesome symptoms (single symptom FSD cases) of 23% in the German general population (*n* = 2379) [13]. The German study examined a smaller sample, slightly younger population and used a stratified sampling strategy. However, we speculate that the difference could also be due to the stricter method used to operationalise the FSD criteria [13]. The study used the BDS Checklist [49] to identify troublesome symptoms, with a stricter operationalisation of single-symptom FSD cases: the respective symptom had to be “severe” and no other “moderate” symptoms could be present in the same organ system. In the original article, the authors suggested further work is needed in categorising severity, citing the bodily distress syndrome methodology [50] as a possible method. Another idea used in the Structured Clinical Interview for DSM-5 (SCID-5) for somatic symptom disorder is to ask patients if and to what extent their symptoms interfere with their daily life, requiring concrete examples to determine if a symptom is severe enough to have diagnostic relevance [51]. Overall, further work is needed to refine the FSD criteria, enhancing specificity for population application while addressing severity and impairment requirements to reliably identify patients. Currently, the criteria for FSD seem overly inclusive. Severity and frequency thresholds for symptoms need to be refined to better identify people who are impaired by their symptoms and would benefit from therapeutic intervention.

### Differences between subgroups of FSD

Current clinical diagnoses are criticised for their inability to incorporate important contextual factors that may contribute to the symptoms [1]. For FSD, it is hypothesised that patients will present with varying specifiers (comorbid conditions and psychological features), which could help guide treatment pathways and new avenues for personalised care. Of those meeting FSD criteria within our sample, a large proportion of people report symptoms from multiple organ systems. We detected weak to moderate differences between subgroups of FSD and non-cases regarding health status, neuroticism, long-term difficulties and healthcare utilisation. Greater differences emerged between those with a FSD system classification (multi or single) compared to those with a single symptom or a non-case—a pattern also found in a previous study [13]. Based on these factors, individuals with FSD with a single symptom were less distinguishable from non-cases, calling into question the need for a diagnostic subgroup with a single persistent and distressing symptom. This study did not assess whether the reported symptoms were ever presented to medical professionals. Patients may be troubled by a persistent troublesome symptom but may not feel the need to seek treatment. So probably not every symptom should be regarded as a sign of a disorder. However, there are also individual severe symptoms, such as fatigue or pain, where patients might benefit from specialised treatment. Unfortunately, the current FSD criteria do not specify the level of severity required for persistent symptoms, beyond describing them as 'troublesome'.

Our results indicated that the majority of individuals in the population who met the criteria for a functional somatic syndrome were most frequently assigned with multi-system FSD, rather than other subgroups. This trend was particularly evident for fibromyalgia and CFS, and to a lesser extent in IBS (Table 2). Our data suggest that many of these patients experience symptoms affecting multiple organ systems, underscoring the potential need for more integrated, multi-system care. Moreover, results could also reflect the earlier discussion that the FSD criteria may need to be more specific. If the intention of the single-system FSD group is to capture syndromes like fibromyalgia, the current framework may not adequately distinguish between single- and multi-system disorders, reinforcing the need for more precise categorisation to ensure appropriate diagnosis and treatment.

### Factors predicting and associated with the FSD classification

The results of the elastic net regression support the inclusion of the specialty-specific dimension of FSD: dysfunctional psychological or behavioural features and the occurrence of a same-system medical condition [1]. A chronic condition in the same organ system predicted FSD, with an odds ratio of 1.8 for musculoskeletal conditions, 1.4 for gastrointestinal conditions, and 1.2 for neurological/other conditions. Previous work has suggested that the originating organ system may not be relevant for the diagnosis of FSD; rather, any physical comorbidity is an important contextual factor [13]. However, in our study, cardiorespiratory conditions were not found to predict FSD. This could be due to the almost equivalent frequency of cardiorespiratory disease across those with multiple system FSD and non-cases (36%), which could indicate that cardiorespiratory disease is less associated with organ-specific persistent troublesome symptoms than other diseases.

In terms of dysfunctional psychological or behavioural features, a concurrent anxiety diagnosis and increased long-term difficulties, such as conflicts with family members or struggles with finances, were associated with a diagnosis of FSD, supporting the importance of investigating these factors when considering a diagnosis. In contrast, co-occurring depression was not associated with FSD. This finding was unexpected, given evidence that depression is usually strongly associated with symptom-based diagnoses [28]. However, depressive and anxiety disorders commonly co-occur [52], and the regression procedure may have selected anxiety as the better predictor. Although this study investigated several potential psychological and behavioural factors, others, such as catastrophising thoughts or avoidance behaviour, are not covered by the Lifelines questionnaires and should be investigated in future studies.

### Limitations

It is important to note that this study used self-report measurements from a population sample only. The optimum way to determine the presence of a FSD would be for a trained clinician to conduct a structured clinical interview while considering differential diagnoses.

In terms of missing data, methods such as multiple imputation were not fully compatible with the elastic net regression, so complete data was used in all statistical analyses. FSD diagnosis was determined based on self-report data, not clinical interviews, and relied on participants actively reporting their symptoms, which could limit the validity of the data, especially when it comes to potential under-reporting of, for example, biomedical chronic comorbidities [53]. In addition, this study partially used cross-sectional data, and therefore, causal explanations cannot be directly inferred. Finally, the duration of six symptoms (difficulty breathing, hot or cold spells, nausea, localised weakness, numbness and dizziness) was indirectly determined by the presence of the symptom on the SCL-90 [54] at separate time points; these symptoms may be susceptible to fluctuations over time.

## Conclusions

Our study provides data-driven evidence for the EURONET-SOMA group’s new diagnostic proposal to classify persistent and troublesome symptoms, i.e., FSD, in a population database. Currently, the criteria for FSD seem overly inclusive. Severity and frequency thresholds for symptoms need to be refined to better identify people who are impaired by their symptoms and would benefit from therapeutic intervention.

Multi-system and single-system FSD is more clinically discernible from non-cases regarding the frequency of comorbidities and psycho-behavioural features. However, this is not the case for single-symptom FSD, which reduces the utility of a single-symptom FSD category. Further work is necessary to define how many symptoms are required for single- and multi-system classification. Subgroup differences could be starker with more stringent subgroup criteria.

Optional specifiers of FSD (comorbid chronic diagnoses and psychological and behavioural factors) are valuable classifiers and can predict a diagnosis of FSD in the population. In clinical practice, these factors could be investigated parallel with treatment to manage the symptoms. Despite our recommendations to refine the criteria, the potential benefits of the FSD classification as an overarching concept to diagnose persistent and troublesome symptoms across medical specialties are promising, and we encourage future research into its development.

## Supplementary Information

Supplementary Material 1. Supplementary file 1 –Table of variables included in analysis. Supplementary File 2 – Table of all chronic conditions by organ system. Supplementary File 3 – Table of symptoms investigated in this study by organ system. Supplementary File 4 – Table of self-reported persistent and troublesome symptoms according to validated questionnaires within the Lifelines sample. Supplementary File 5 – Bar graph depicting self-reported bothersome persistent symptom frequencies. Supplementary File 6 – Sankey diagram illustrating the flow of the population divided by subgroups of FSD and the relative proportions of comorbidities.

## Data Availability

Data may be obtained from a third party and are not publicly available. Researchers can apply to use the Lifelines data used in this study. More information about how to request Lifelines data and the conditions of use can be found on their website (https://www.lifelines-biobank.com/researchers/working-with-us).

## Abbreviations

FSD: Functional somatic disorder
SSD: Somatic symptom disorder
BDD: Bodily distress disorder
IBS: Irritable bowel syndrome
CFS: Chronic fatigue syndrome
BDS: Bodily distress syndrome
