# Supplementary material for: Empirical assessment of functional somatic disorder (FSD): frequency, applicability, and diagnostic refinement in a population-based sample
Source: BMC Med. 2025 Apr 14;23:221. doi: 10.1186/s12916-025-04042-w (PMC11998262; doi:10.1186/s12916-025-04042-w)

## Supplementary File 1 –Table of variables included in analysis

| Items | Analysis |  | Values |
| --- | --- | --- | --- |
| **Research specific** |  |  |  |
| FSD Case | Regression | Binary | 0 = Non-case  1 = Participant qualifies for at least one FSD defined symptom |
| Subgroup of FSD criteria | Kruskal-Wallis  Chi-square | Categorical | 0 = Non-case  1 = Single symptom  2 = Single system  3 = Multi-system |
| Number of symptoms meeting FSD symptom classification | Kruskal-Wallis | Continuous | Sum score 0 to 25 |
| Number of FSD organ systems a participant experiences FSD symptoms from | - | Categorical | 1 = One system  2 = Two systems  3 = Three systems  4 = Four systems |
| Functional syndrome criteria met   1. Chronic Fatigue Syndrome (CFS) 2. Irritable Bowel Syndrome (IBS) 3. Fibromyalgia (FM) | Chi-square Regression | Categorical | 1 = Participant found to reliably meet criteria  0 = Participant does not reliably meet criteria |
|  | - | Continuous | Count, number of functional syndromes met (0 – 3) |
| Participant reports a condition from a particular bodily system:   1. Cardio/pulmonary 2. Musculoskeletal 3. Gastrointestinal   Neurological/other | Regression Chi-square | Binary | 1 = comorbidity present  0 = no comorbidity present |
| Participant possesses a same-system comorbidity according to FSD criteria:   1. Cardio/pulmonary 2. Musculoskeletal 3. Gastrointestinal   Neurological/other | - | Binary | 1 = comorbidity present  0 = no comorbidity present |
| Psychological features   1. Any anxiety 2. Any depression | Regression  Chi-square | Binary | 1 = concurrent diagnosis via mini interview  0 = no concurrent diagnosis |
| **General information and Demographics** |  |  |  |
| Sex | Chi-square  Regression | Binary | 0 = Male  1 = Female |
|  |  |  |  |
| Age | Regression  Kruskal-Wallis | Continuous | Years, range 0-50+ |
| Ethnicity | Chi-square | Categorical | 1 = white/eastern and western Europe  2 = white/mediterranean or arabic  3 = black  4 = asian  5 = other |
| How long have you been in your current relationship? | Kruskal-Wallis  Regression | Continuous | Years, range 0-50+ |
| Living situation |  |  |  |
| How many people are living in your house? Please note: we refer to the house where you live most days of the week. | Kruskal-Wallis  Regression | Continuous | Count, range 0-6+ |
| What is the highest level of education you have finished?   1. Low (no education, primary education, lower or preparatory vocational education, lower general secondary education) 2. Medium (intermediate vocational education or apprenticeship, higher general senior secondary education or pre-university secondary education) 3. Higher (higher vocational education, university) | Kruskal-Wallis  Regression | Categorical | 1 = low education  2 = medium  education  3 = high education |
| How many hours do you do paid work on average? | Kruskal-Wallis  Regression | Continuous | Hours, range 0-60+ |
| **Psychological features** |  |  |  |
| **Loneliness**  Loneliness Scale - De Jong Gierveld, J. (1989)  Measures subjective social isolation | Kruskal-Wallis  Regression | Continuous | Sum score, range 0 – 11 (Cut offs: 0-2 not lonely, 3-8 moderately lonely, 9-11 strongly lonely) |
|  |  |  |  |
| Long-term difficulties |  |  |  |
| Childhood Trauma Questionnaire (CTQ) subtype 2b   1. Emotional neglect 2. Physical abuse 3. Sexual abuse 4. Emotional neglect 5. Physical neglect 6. No abuse   Total CTQ score (sum of subscales 1-5)  Cut off score >35. | -  Kruskal-Wallis  Regression | Ordinal  Continuous | Sum, range 0 – 4  “”  “”  “”  “”  Sum, range 0 - 3  Sum score, range 25 - 125 |
| LDI measures difficulty and stress in the last 12 months including 12 aspects of life: housing, work, social relationships, free time, finances, health, school/study and religion.  0=not stressful, 1= slightly stressful, 2=very stressful | Regression  Kruskal-Wallis | Continuous | Sum score, 0 to possible 24 |
| LTE measures the occurrence stressful life events in the past year   - 12 major categories of stressful life events that were selected for their established long-term consequences | Regression  Kruskal-Wallis | Continuous | Sum of the  item scores for each category, 0 to possible 12 |
| Personality (NEO-PI-R) |  |  |  |
| Neuroticism scale | Regression Kruskal-Wallis | Continuous | Imputed sum score 0 - 100 |
| **Healthcare Utilisation** |  |  |  |
| Number of healthcare providers visited in the past 12 months   1. Psychologist 2. Physiotherapist 3. Occupational 4. Obstetrician 5. Mental health care 6. Indication center 7. Home care 8. General Practitioner 9. GP center (not GP but another practitioner) 10. Dietician 11. Dentist 12. Company doctor 13. Alternative practice 14. Addiction care provider CAD 15. Addiction care for the Northern Netherlands VNN 16. Specialist care 17. Public health care 18. Speech therapist 19. Remedial therapist 20. Welfare   (Plus variable indicates no healthcare contact in the past year = 0) | Regression  Kruskal-Wallis | Continuous | Count, number of specialists visited, range 0 to 20 (removed no healthcare) |
| **Physical Functioning and Health Status** |  |  |  |
| EQ-5D-5L Visual Analogue Scale of health status | Regression Kruskal-Wallis | Continuous | 0 to 100 |

## Supplementary File 2 – Table of all chronic conditions by organ system

| Cardiopulmonary | Gastrointestinal | Musculoskeletal | Neurological |
| --- | --- | --- | --- |
| Heart attack | Coeliac disease | Osteoarthritis | Dementia |
| Atherosclerosis | Crohns disease | Rheumatoid Arthritis | Epilepsy |
| Heart valve problems | Gallstones |  | Migraine |
| Pulmonary embolism | Ulcers |  | Multiple Sclerosis |
| Cardio vascular disease | Hepatitis |  | Parkinson's |
| COPD | Liver cirrhosis |  | Stroke |
| Asthma | Ulcerative colitis |  |  |

## Supplementary File 3 – Table of symptoms investigated in this study by organ system

| Cardiopulmonary | Gastrointestinal | Musculoskeletal | Neurological |
| --- | --- | --- | --- |
| Hot or cold spells | Frequent loose bowel movements | Pains in arms or legs | Concentration difficulties |
| Chest pain | Abdominal pains | Muscular aches or pains | Excessive fatigue |
| Difficulty breathing | Feeling bloated/full of gas/distended | Other pain | Headache |
|  | Hard stools | Pains in the joints | Impairment of memory |
|  | Constipation | Localized weakness | Dizziness |
|  | Burning sensation of the upper part of stomach/epigastrium | Back ache |  |
|  | Unpleasant feeling/bloating after meals | Unpleasant numbness or tingling sensations |  |
|  | Unable to eat complete meal |  |  |
|  | Nausea |  |  |

## Supplementary File 4 – Table of self-reported persistent and troublesome symptoms according to validated questionnaires within the Lifelines sample

| FSD Symptoms | Source | Duration | Troublesome impact of symptoms | Symptom present | Not present | Total respondents | Percentage of respondents (valid percent) | Percentage of total sample |
| --- | --- | --- | --- | --- | --- | --- | --- | --- |
| Chest pain | WPI | Required “six months or longer” | WPI response “quite a bit”, “a lot”, or “very much” | 1601 | 86037 | 87638 | 1.83% | 1.80% |
| Difficulty breathing | SCL-90 | Presence of symptom at timepoint 1C and 2A (± 10 months) | Moderate to severely impacting symptoms (score of 2 or greater) | 83 | 68839 | 68922 | 0.12% | 0.09% |
| Hot or cold spells | SCL-90 | Presence of symptom at timepoint 1C and 2A (± 10 months) | Moderate to severely impacting symptoms (score of 2 or greater) | 567 | 62576 | 63143 | 0.90% | 0.64% |
| Frequent loose bowels | ROME-III | Required longer than six months | ROME required symptoms present “often”, “most of the time”, or “always” on three days per month OR hampering normal activities “quite a bit”, “a lot”, or “very much” | 4947 | 30764 | 35711 | 13.85% | 5.56% |
| Abdominal pain | ROME-III or WPI | Required “six months or longer” | WPI response “quite a bit”, “a lot”, or “very much” OR ROME required symptoms present “often”, “most of the time”, or “always” on three days per month OR hampering normal activities “quite a bit”, “a lot”, or “very much” | 21372 | 26294 | 47666 | 44.84% | 24.03% |
| Bloated | ROME-III | Required longer than six months | ROME required symptoms present “often”, “most of the time”, or “always” on three days per month OR hampering normal activities “quite a bit”, “a lot”, or “very much” | 21372 | 31291 | 52663 | 40.58% | 24.03% |
| Constipation | ROME-III | Required longer than six months | ROME required symptoms present “often”, “most of the time”, or “always” on three days per month OR hampering normal activities “quite a bit”, “a lot”, or “very much” | 4241 | 83203 | 87444 | 4.85% | 4.77% |
| Nausea | SCL-90 | Presence of symptom at timepoint 1C and 2A (± 10 months) | Moderate to severely impacting symptoms (score of 2 or greater) | 170 | 62708 | 62878 | 0.27% | 0.19% |
| Upper stomach burning | ROME-III | Required longer than six months | ROME required symptoms present “often”, “most of the time”, or “always” on three days per month OR hampering normal activities “quite a bit”, “a lot”, or “very much” | 602 | 14011 | 14613 | 4.12% | 0.68% |
| Unpleasant after meals | ROME-III | Required longer than six months | ROME required symptoms present “often”, “most of the time”, or “always” on three days per month OR hampering normal activities “quite a bit”, “a lot”, or “very much” | 68 | 21031 | 21099 | 0.32% | 0.08% |
| Unable to complete meals | ROME-III | Required longer than six months | ROME required symptoms present “often”, “most of the time”, or “always” on three days per month OR hampering normal activities “quite a bit”, “a lot”, or “very much” | 1624 | 10162 | 11786 | 13.78% | 1.83% |
| Limb pain | WPI | Required “six months or longer” | WPI response “quite a bit”, “a lot”, or “very much” | 6806 | 55975 | 62781 | 10.84% | 7.65% |
| Muscle pain | CDC | Required “longer than 6 months” | CDC symptoms occurred “a few times a week” or “everyday” | 10221 | 36509 | 46730 | 21.87% | 11.49% |
| Neck pain | WPI | Required “six months or longer” | WPI response “quite a bit”, “a lot”, or “very much” | 5676 | 82389 | 88065 | 6.45% | 6.38% |
| Joint pain | CDC or WPI | Required “six months or longer” | WPI response “quite a bit”, “a lot”, or “very much” OR CDC symptoms occurred “a few times a week” or “everyday” | 13221 | 18671 | 31892 | 41.46% | 14.86% |
| Localised weakness | SCL-90 | Presence of symptom at timepoint 1C and 2A (± 10 months) | Moderate to severely impacting symptoms (score of 2 or greater) | 577 | 68349 | 68926 | 0.84% | 0.65% |
| Back ache | WPI | Required “six months or longer” | WPI response “quite a bit”, “a lot”, or “very much” | 7201 | 80440 | 87641 | 8.22% | 8.10% |
| Numbness | SCL-90 | Presence of symptom at timepoint 1C and 2A (± 10 months) | Moderate to severely impacting symptoms (score of 2 or greater) | 616 | 68437 | 69053 | 0.89% | 0.69% |
| Poor concentration | CDC | Required “longer than 6 months” | CDC symptoms occurred “a few times a week” or “everyday” | 7562 | 23252 | 30814 | 24.54% | 8.50% |
| Fatigue | CIS cut off score | Additional item question investigating fatigue duration required over six months | CIS severity using the 35-point recommended cut-off | 21054 | 66872 | 87926 | 23.95% | 23.67% |
| Headache | CDC | Required “longer than 6 months” | CDC symptoms occurred “a few times a week” or “everyday” | 4905 | 37559 | 42464 | 11.55% | 5.51% |
| Impaired memory | CDC | Required “longer than 6 months” | CDC symptoms occurred “a few times a week” or “everyday” | 7785 | 24933 | 32718 | 23.79% | 8.75% |
| Dizziness | SCL-90 | Presence of symptom at timepoint 1C and 2A (± 10 months) | Moderate to severely impacting symptoms (score of 2 or greater) | 180 | 7872 | 8052 | 2.24% | 0.20% |
| Post exertional malaise | CDC | Required “longer than 6 months” | CDC symptoms occurred “a few times a week” or “everyday” | 8212 | 22166 | 30378 | 27.03% | 9.23% |
| Unrefreshing sleep | CDC | Required “longer than 6 months” | CDC symptoms occurred “a few times a week” or “everyday” | 15757 | 36040 | 51797 | 30.42% | 17.71% |
| Note: for additional detail on all measure the Lifelines implements across the cohort study see the Wiki (https://wiki.lifelines.nl/doku.php) | | | | | | | | |

## Supplementary File 5 – Bar graph depicting self-reported bothersome persistent symptom frequencies within the total lifelines sample

## Supplementary File 6 – Sankey diagram illustrating the flow of the population divided by subgroups of FSD and the relative proportions of comorbidities


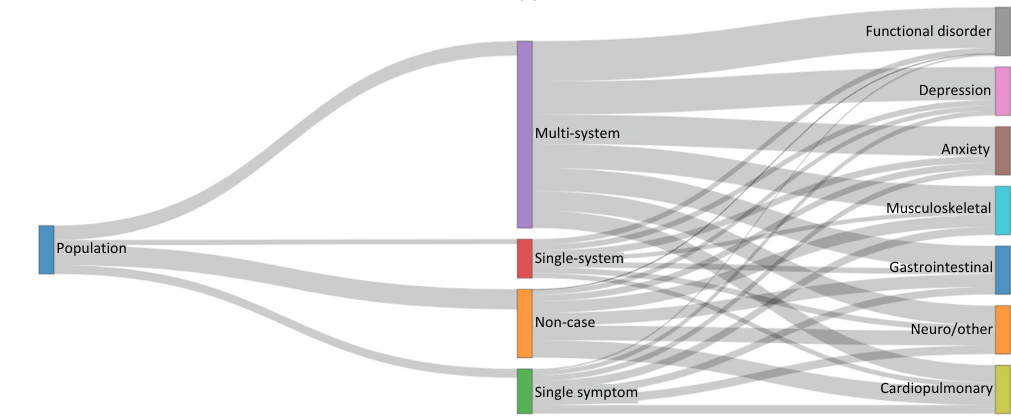

Supplement: Supplementary file 1 — Supplementary Material 1. Supplementary file 1 –Table of variables included in analysis. Supplementary File 2 – Table of all chronic conditions by organ system. Supplementary File 3 – Table of symptoms investigated in this study by organ system. Supplementary File 4 – Table of self-reported persistent and troublesome symptoms according to validated questionnaires within the Lifelines sample. Supplementary File 5 – Bar graph depicting self-reported bothersome persistent symptom frequencies. Supplementary File 6 – Sankey diagram illustrating the flow of the population divided by subgroups of FSD and the relative proportions of comorbidities. [file 12916_2025_4042_MOESM1_ESM.docx]
